# Regional disparities in infant mortality in Canada: a reversal of egalitarian trends

**DOI:** 10.1186/1471-2458-9-4

**Published:** 2009-01-07

**Authors:** K S Joseph, Ling Huang, Susie Dzakpasu, Catherine McCourt

**Affiliations:** 1 Perinatal Epidemiology Research Unit, Department of Obstetrics & Gynaecology, Dalhousie University and the IWK Health Centre, Halifax, Canada; 2 Perinatal Epidemiology Research Unit, Department of Pediatrics, Dalhousie University and the IWK Health Centre, Halifax, Canada; 3 Maternal and Infant Health Section, Health Surveillance and Epidemiology Division, Public Health Agency of Canada, Ottawa, Canada

## Abstract

**Background:**

Although national health insurance plans and social programs introduced in the 1960s led to reductions in regional disparities in infant mortality in Canada, it is unclear if such patterns prevailed in the 1990s when the health care and related systems were under fiscal duress. This study examined regional patterns of change in infant mortality in Canada in recent decades.

**Methods:**

We analysed regional changes in crude infant mortality rates and in infant mortality rates among live births with a birth weight ≥ 500 g and ≥ 1,000 g in Canada from 1945 to 2002. Associations between baseline infant mortality rates in the provinces and territories (e.g., in 1985–89) and the change observed in infant mortality rates over the subsequent period (e.g., between 1985–89 and 1995–99) were assessed using Spearman's rank correlation coefficient. Trends in regional disparities were also assessed by calculating period-specific rate ratios between provinces/territories with the highest versus the lowest infant mortality.

**Results:**

Provincial/territorial infant mortality rates in 1945–49 were not correlated with changes in infant mortality over the next 10 years (rho = 0.01, P = 0.99). However, there was a strong negative correlation between infant mortality rates in 1965–69 and the subsequent decline in infant mortality (rho = - 0.85, P = 0.002). Provinces/territories with higher infant mortality rates in 1965–69 (Northwest Territories 64.7 vs British Columbia 20.7 per 1,000 live births) experienced relatively larger reductions in infant mortality between 1965–69 and 1975–79 (53.7% decline in the Northwest Territories vs a 36.6% decline in British Columbia). This pattern was reversed in the more recent decades. Provinces/territories with higher infant mortality rates ≥ 500 g in 1985–89 experience relatively smaller reductions in infant mortality between 1985–89 and 2000–02 (rho = 0.82, P = 0.004). The infant mortality ≥ 500 g rate ratio (contrasting the province/territory with the highest versus lowest infant mortality) was 3.2 in 1965–69, 2.4 in 1975–79, 2.2 in 1985–89, 3.1 in 1995–99 and 4.1 in 2000–02.

**Conclusion:**

Fiscal constraints in the 1990s led to a reversal of provincial/territorial patterns of change in infant mortality in Canada and to an increase in regional health disparities.

## Background

National health insurance programs have proved to be one of the most important public health initiatives in industrialized countries since the second World War. In Canada, the introduction of insurance programs for hospital and related services (Hospital and Diagnostic Services Act, 1957) and for physician services (Medical Care Act, 1966 [1]) was a consequence of concerns about the prohibitive costs of health care and unequal access to essential health services. Provinces and territories received federal financial support if their health care systems met the conditions of public administration, comprehensiveness, universality, portability and accessibility [1,2].

Besides creating an egalitarian system of health service delivery and ushering improvements in public health, this insurance program (along with federal equalization payments which support social programs and education) led to a reduction in health disparities across Canada. Dzakpasu et al [3] showed that Canadian provinces and territories with the highest infant mortality rates in the pre-Medicare era showed the largest declines in infant mortality in subsequent years. This phenomenon runs counter to trends prevailing globally (labelled the Matthew effect [4,5]). International comparisons of infant mortality show countries with low rates of infant mortality achieving the largest subsequent declines [3,5].

Idealistic rhetoric notwithstanding, the health care system in Canada suffered a serious fiscal crisis in the 1990s as the federal government addressed the problem of a soaring budget deficit by reducing its financial commitment to provincial health insurance plans and related social programs [6,7]. Federal transfers declined from $8.2 billion in 1992 to $6.3 billion in 1996 (then increased to $8.8 billion in 2001). Public health care expenditure declined from 74.1% of the total health care expenditure in 1992 to 70.8% in 1996 and was 70.1% in 2001 [7]. This had a profound effect on hospital and related sectors with a substantial reduction in hospital care, despite the continued pressures of population aging and population growth. Spending on hospitals and physicians which comprised 40.6% and 15.6% of health care spending, respectively, in 1987 fell to 33.6% and 14.5% of health care spending, respectively, in 1997 [6]. Social programs were similarly affected.

We carried out a study to examine the effect of these changes in public health funding in the 1990s, on regional disparities in health status. We used the infant mortality rate to assess health status, as this index is a good indicator of population health and is susceptible to changes in health care delivery and social programs. In Canada, approximately two-thirds of infant mortality is neonatal (and potentially impacted by health care funding), while one-third is post-neonatal [8] (and potentially susceptible to funding for social programs and health care services in the community).

## Methods

Data on live births and infant deaths were obtained from the publications [9-18], vital statistics files and birth cohort files of Statistics Canada [19]. Five years of infant mortality data were used to define period rates in order to lend stability to estimates from the smaller provinces and territories. In order to obtain a comprehensive perspective, we analysed patterns of change in infant mortality rates from 1945–49 to 2000–02.

Newfoundland was excluded from the analysis because data from this province were not available for all the years under study and Ontario was excluded because of concerns regarding data quality [20,21]. Ontario data were included in supplementary analyses. For the purposes of this analysis, data from Nunavut (created from the Northwest Territories in 1999) were combined with that for the Northwest territories.

Differences in the registration of live births at the borderline of viability (live births with a birth weight < 500 g or gestational age < 22 weeks) can bias regional and temporal comparisons [22-26]. To address this problem, the World Health Organization recommends that international comparisons of infant mortality be restricted to live births with a birth weight ≥ 1,000 g [27]; the Canadian Perinatal Surveillance System publishes information on interprovincial/territorial infant mortality rates among live births with a birth weight ≥ 500 g and ≥ 1,000, in addition to crude infant mortality rates [8] for this reason. Such information on birth weight-specific infant mortality in Canada was only available from 1985 onwards. Provincial/territorial rates of infant mortality among live births ≥ 500 g for 1965–69 and 1975–79 were obtained by assuming that all infants with a birth weight < 500 g did not survive infancy. This assumption is not untenable because infant mortality rates among live births < 500 g are very high (943 per 1,000 live births in 1997–99 [8]).

Although the magnitude of absolute declines in infant mortality is important from a public health standpoint, we chose to focus on the relative change in infant mortality as this comparative measure was deemed to be unmodified by the background rate of infant mortality [28]. Thus a decline in the infant mortality rate from 50 per 1,000 live births to 25 per 1,000 live births (50% decline) was deemed equivalent to a decline in infant mortality from 20 per 1,000 live births to 10 per 1,000 live births (50% decline) even though the absolute decline was larger in the former instance. Potential associations between baseline infant mortality rates in the provinces and territories (e.g., in 1985–89) and the change observed in infant mortality rates over the subsequent period (e.g., between 1985–89 and 1995–99) were assessed using Spearman's rank correlation coefficient (rho). These correlation coefficients were complemented with 2-sided P values.

We also examined the difference between the provinces with the highest versus the lowest infant mortality rates. As with previous analyses, these contrasts were made in relative terms (infant mortality rate ratios) and carried out separately for crude rates, infant deaths ≥ 500 g and infant deaths ≥ 1,000 g. Infant mortality rate ratios expressing disparities were also calculated by contrasting the 2 provinces/territories with the highest infant mortality versus the 2 provinces/territories with the lowest infant mortality. 95% confidence intervals (CIs) were estimated for these infant mortality rate ratios to express the precision of the estimates.

## Results

**Figure 1:**
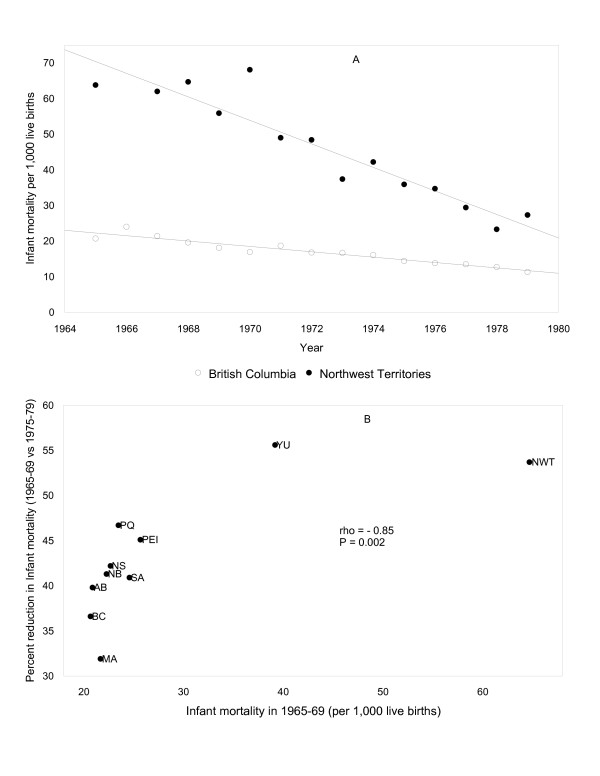
**Crude infant mortality rates in 2 selected provinces between 1965 and 1979 (Figure 1A) and the relationship between infant mortality in 1965–69 and the change between 1965–69 and 1975–79 in the provinces and territories of Canada (Figure 1B)**. Abbreviations used: PEI Prince Edward Island; NS Nova Scotia; NB New Brunswick; PQ Quebec; MA Manitoba; SA Saskatchewan; AB Alberta; BC British Columbia; YU Yukon; NWT North West Territories.

Table 1 shows infant mortality rates in the provinces/territories of Canada in 1965–69 and changes in rates between 1965–69 and 1975–79 (Table 1). A significant negative correlation was observed between the infant mortality rate in 1965–69 and the decline in infant mortality over the subsequent decade i.e., provinces with high infant mortality rates in 1965–69 had larger reductions in infant mortality between 1965–69 and 1975–79. For instance, North West Territories, which had a much higher infant mortality in 1965–69 than British Columbia, experienced a much larger decline in infant mortality over the next ten years. Inclusion of Ontario data did not alter this correlation materially. Figure 1A shows the infant mortality rates by year in 2 selected provinces between 1965 and 1979 and Figure 1B shows the relationship between infant mortality in 1965–69 and the change between 1965–69 and 1975–79.

**Table 1:** Crude infant mortality rates in the provinces and territories of Canada in 1965–69 and changes in infant mortality rates between 1965–69 and 1975–79.

Province/Territory*	Crude infant mortality rate	
	
	in 1965–69 per 1,000 live births (rank)	% change 1975–79 vs 1965–69 (rank)
British Columbia	20.7 (1)	-36.6 (9)
Alberta	20.9 (2)	-39.8 (8)
Manitoba	21.7 (3)	-31.9 (10)
New Brunswick	22.3 (4)	-41.3 (6)
Nova Scotia	22.7 (5)	-42.2 (5)
Quebec	23.5 (6)	-46.7 (3)
Saskatchewan	24.6 (7)	-40.9 (7)
Prince Edward Island	25.7 (8)	-45.1 (4)
Yukon	39.2 (9)	-55.6 (1)
Northwest Territories	64.7 (10)	-53.7 (2)

Spearman's rank correlation coefficient = -0.85, P = 0.002 i.e., provinces with higher infant mortality rates in 1965–69 experienced larger reductions in infant mortality over the next decade and this association was statistically significant.

* The infant mortality in Ontario in 1965–69 was 19.4 per 1,000 live births and this declined by 40.2% between 1965–69 and 1975–79. Inclusion of Ontario data gave essentially the same results (correlation coefficient = -0.84, P = 0.001).

Table 2 shows the provincial and territorial infant mortality rates among live births with a birth weight ≥ 500 g in 1985–89 and the change in infant deaths ≥ 500 g between 1985–89 and 1995–99. There was a non-significant positive correlation between infant mortality ≥ 500 g in 1985–89 and the change over the subsequent 10 years. Provincial and territorial infant mortality rates in 1985–89 among live births with a birth weight ≥ 1,000 g and the change in infant deaths up to 1995–99 are presented in Table 3. There was a non-significant positive correlation between infant mortality ≥ 1,000 g in 1985–89 and the change between 1985–89 and 1995–99.

**Table 2:** Infant mortality rate among live births ≥ 500 g in the provinces and territories of Canada in 1985–89 and changes in infant mortality rates between 1985–89 vs 1995–99.

Province/Territory*	Infant mortality rate ≥ 500 g	
	
	in 1985–89 per 1,000 live births (rank)	% change 1995–99 vs 1985–89 (rank)
Prince Edward Island	6.2 (1)	-18.3 (8)
Nova Scotia	6.5 (2)	-38.7 (2)
Quebec	6.5 (3)	-33.4 (5)
New Brunswick	7.1 (4)	-37.5 (3)
British Columbia	7.7 (5)	-46.4 (1)
Manitoba	7.9 (6)	-25.3 (7)
Alberta	8.0 (7)	-37.2 (4)
Saskatchewan	8.4 (8)	-13.3 (9)
Yukon	9.9 (9)	-30.0 (6)
Northwest Territories	13.8 (10)	-9.5 (10)

Spearman's rank correlation coefficient = 0.45, P = 0.19 i.e., provinces with higher infant mortality rates in 1985–89 experienced smaller reductions in infant mortality over the next decade but this association was not statistically significant.

* The infant mortality in Ontario among live births ≥ 500 g in 1985–89 was 6.4 per 1,000 live births and this declined by 21.5% between 1985–89 and 1995–99. Inclusion of Ontario data gave similar results (correlation coefficient = 0.26, P = 0.45).

**Table 3:** Infant mortality rate among live births ≥ 1,000 g in the provinces and territories of Canada in 1985–89 and changes in infant mortality rates between 1985–89 vs 1995–99.

Province/Territory*	Infant mortality rate ≥ 1,000 g	
	
	in 1985–89 per 1,000 live births (rank)	% change 1995–99 vs 1985–89 (rank)
Prince Edward Island	4.6 (1)	-29.6 (7)
Nova Scotia	5.0 (2)	-39.0 (5)
Quebec	5.1 (3)	-39.4 (3)
New Brunswick	5.8 (4)	-41.0 (2)
British Columbia	5.8 (5)	-46.7 (1)
Alberta	6.0 (6)	-39.0 (4)
Manitoba	6.3 (7)	-30.4 (6)
Saskatchewan	6.3 (8)	-13.6 (9)
Yukon	8.7 (9)	-25.3 (8)
Northwest Territories	12.1 (10)	-6.6 (10)

Spearman's rank correlation coefficient = 0.56, P = 0.09 i.e., provinces with higher infant mortality rates among live births ≥ 1,000 g in 1985–89 experienced smaller reductions in infant mortality over the next decade but this association was not statistically significant.

* The infant mortality in Ontario among live births ≥ 1,000 g in 1985–89 was 5.2 per 1,000 live births and this declined by 21.9% between 1985–89 and 1995–99. Inclusion of Ontario data gave a similar result (correlation coefficient = 0.45, P = 0.17).

**Figure 2:**
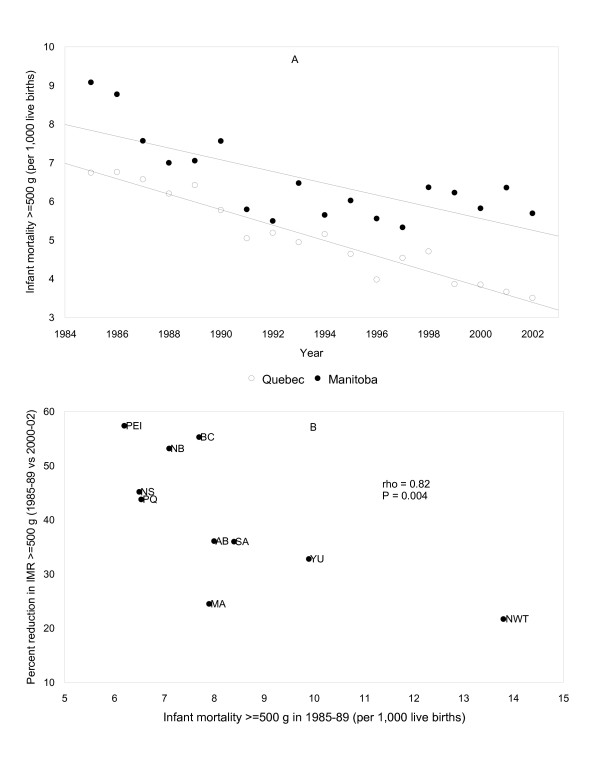
**Infant mortality rates ≥ 500 g in 2 selected provinces between 1985 and 2002 (Figure 2A) and the relationship between infant mortality in 1985–89 and the change between 1985–89 and 2000–02 in the provinces and territories of Canada (Figure 2B)**. Abbreviations used: PEI Prince Edward Island; NS Nova Scotia; NB New Brunswick; PQ Quebec; MA Manitoba; SA Saskatchewan; AB Alberta; BC British Columbia; YU Yukon; NWT North West Territories.

All period contrasts are presented in Table 4. There was no correlation in baseline infant mortality and the subsequent change in infant mortality prior to the advent of national health insurance (i.e., 1945–49 vs 1955–59). The correlation became strongly negative following the introduction of national health insurance (1965–69 vs 1975–79) then weakened (1975–79 vs 1985–89) before turning positive in the 1990s and beyond (1985–89 vs 1995–99 and 2000–02). This positive correlation was most strongly evident in the relationship between infant mortality ≥ 500 g in 1985–89 and the change between 1985–89 and 2000–02 (Figure 2). Infant mortality rates among live births ≥ 1,000 g in 1985–89 were also significantly and positively correlated with reductions between 1985–89 and 2000–02.

**Table 4:** Correlation between infant mortality rates in the provinces and territories of Canada and subsequent change in infant mortality rates.

Index	Baseline period	Change to	Spearman's correlation coefficient (rho)	P value
Crude infant mortality rate				
	1945–49	1955–59	0.01	0.99
	1955–59	1965–69	-0.55	0.10
	1965–69	1975–79	-0.85	0.002
	1965–69	1985–89	-0.90	<0.001
	1975–79	1985–89	-0.25	0.49
	1985–89	1995–99	0.20	0.58
	1985–89	2000–02	0.30	0.40
	1995–99	2000–02	-0.33	0.35
Infant mortality rate among live births ≥ 500 g*				
	1965–69	1975–79	-0.84	0.002
	1975–79	1985–89	-0.36	0.31
	1985–89	1995–99	0.46	0.19
	1985–89	2000–02	0.82	0.004
	1995–99	2000–02	0.03	0.93
Infant mortality rate among live births ≥ 1,000 g				
	1985–89	1995–99	0.56	0.09
	1985–89	2000–02	0.77	0.009
	1995–99	2000–02	0.13	0.73

* Data for 1985 to 2002 based on birth cohort data. Data for 1965–69 and 1975–79 assumes that all live births < 500 g died in infancy (see text). Estimates of the number of live births < 500 g in the Northwest Territories in 1965–67 were extrapolated from data from the Northwest Territories in 1968–69 [12,13].

**Figure 3:**
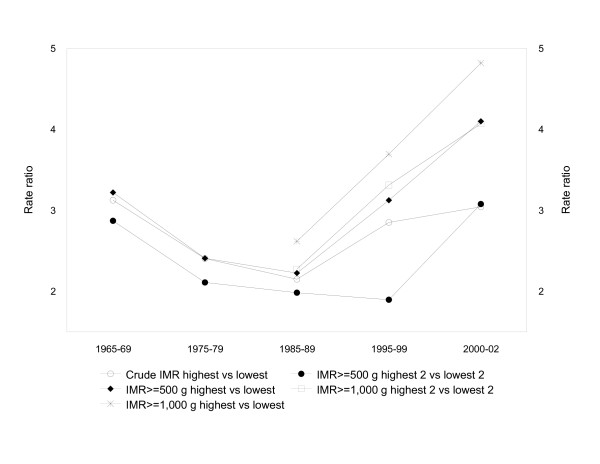
**Rate ratios contrasting crude and birth weight-specific infant mortality rates (≥ 500 g and ≥ 1,000 g) in the provinces/territories with the highest versus the lowest rates, Canada 1965–69 to 2000–02**.

The rate ratio expressing the regional disparity in infant mortality ≥ 500 g between the province/territory with the highest infant mortality vs the province/territory with the lowest infant mortality was 3.2 in 1965–69. This ratio declined to 2.4 in 1975–79 and 2.2 in 1985–89, before rising to 3.1 in 1995–99 and 4.1 in 2000–02. The rate ratio expressing the infant mortality rate ≥ 1,000 g between the provinces/territories with the highest vs lowest infant mortality was 2.6, 3.7 and 4.8 in 1985–89, 1995–99 and 2000–02, respectively. Figure 3 shows temporal trends in regional disparities including contrasts of the 2 provinces/territories with the highest infant mortality vs the 2 provinces/territories with lowest rates. These rate ratios were 2.9 (95% CI 2.6–3.2) in 1965–69, 2.1 (95% CI 1.8–2.4) in 1975–79, 2.0 (95% CI 1.6–2.4) in 1985–89, 1.9 (95% CI 1.7–2.1) in 1995–99 and 3.1 (95% CI 2.2–4.4) in 2000–02 for infant deaths ≥ 500 g. For infant deaths ≥ 1,000 g these mortality rate ratios were 2.3 (95% CI 1.8–2.8) in 1985–89, 3.3 (95% CI 2.7–4.1) in 1995–99 and 4.1 (95% CI 2.7–6.1) in 2000–02.

Supplementary analyses showed that exclusion of the Yukon and the Northwest Territories (the 2 regions with the highest mortality rates in most periods) did not alter the results generally. After this study was completed, birth cohort data for 2003 became available and additional analyses showed that the correlation between crude infant mortality rates in 1985–89 and change between 1985–89 and 2003 was 0.17 (P = 0.63); that between infant mortality ≥ 500 g and change between 1985–89 and 2003 was 0.21 (P = 0.56); and that between infant mortality ≥ 1,000 g and change between 1985–89 and 2003 was also 0.21 (P = 0.56).

## Discussion

Our study shows that provinces and territories with a higher infant mortality rate in 1965–69 experienced larger declines in infant mortality over the next decade. This pattern was very different from the pattern observed in the provinces and territories between 1985–89 and subsequent years. Correlations between infant mortality rates in 1985–89 and 2000–02 among live births ≥ 500 g and ≥ 1,000 g suggest that the fiscal constraints of the 1990s contributed to an increase in health disparities in Canada. These results were corroborated by a second analysis of regional disparities which showed an increasing infant mortality rate ratio between provinces/territories with the highest vs the lowest infant mortality rates.

There was a non-significant positive correlation between infant mortality rates in the provinces/territories in 1985–89 and the change in infant mortality between 1985–89 and 1995–99. The correlation was stronger when infant mortality rates were examined among live births with a birth weight ≥ 500 g and ≥ 1,000 g. Similarly, correlations between infant mortality rates in 1985–89 and the change in infant mortality between 1985–89 and 2000–02 among live births ≥ 500 g and ≥ 1,000 g were positive and significant, though that based on the crude infant mortality rate was not (Table 3). As mentioned, birth weight-specific analyses of infant mortality are preferable [22-26] since they eliminate biases due to regional and temporal variations in birth registration.

The reductions in disparities observed between 1965–69 and 1975–79 did not come at the expense of the provinces with lower infant mortality rates. Provinces such as Ontario and British Columbia with the lowest infant mortality rates in 1965–69 (ranks 1 and 2), continued to have the lowest rates in 1975–79 (ranks 1 and 5) despite having lower rates of decline between 1965–69 and 1975–79 relative to other regions. Such patterns of change are required if regional disparities in infant mortality are to be eliminated. On the other hand, the pattern of change observed between 1985–89 and 2000–02 tends to exacerbate regional disparities in health status (evident in the infant mortality rate ratios between regions). Attenuation of the correlations in the contrast up to 2003 occurred possibly because of the restoration of federal transfers.

The strong negative correlation observed soon after the introduction of Medicare (1965–69 vs 1975–79) had diminished to a weak negative correlation (1975–79 vs 1985–89) before reversing direction and becoming a positive correlation more recently. It appears that the commitment to reducing regional disparities suffered a gradual decline over several decades. It may therefore be unfair to attribute the reversal of the trends in regional disparities solely to federal fiscal policy in the 1990s.

The ecologic design and our conclusions focus on the single issue of changes in health care funding for the provincial/territorial health insurance plans and related social programs. It is possible that other unrelated factors which we did not consider were responsible for the differences observed. Thus much of the reduction in infant mortality, especially neonatal mortality, would have occurred due to advances in medical technologies (including Rh immune globulin, cesarean delivery, neonatal intensive care, antenatal steroid prophylaxis, surfactant, and assisted ventilation) and the regionalization of perinatal care [29,30]. However, any proposed factor would have had to cause differential effects on health status across regions and over time in order to yield the observed patterns of changes. Another limitation was our inability to include all provinces and territories of Canada in our analysis because of data constraints. We did carry out supplementary analyses using available data for Ontario, however, and these did not change our findings. The number of statistical tests carried out in this study (32 P values in Table 4) could mean that some of our significant P values may have been < 0.05 by chance i.e., due to multiple hypothesis testing. We do not believe this potential limitation represents a serious issue because our hypothesis was well defined and our conclusions were based on patterns of change rather than a single significant comparison.

## Conclusion

In conclusion, our study shows that the health care system and related social programs in Canada, introduced in the late 1950s and 1960s, were responsible for substantially reducing regional disparities in infant mortality. Whereas the system continues to have a beneficial effect and Canadian infant mortality rates are among the lowest in the world, the original commitment to egalitarian goals diminished in the 1990s, at least as judged by the pattern of reductions in infant mortality between 1985–89 and 1995–99 and between 1985–89 and 2000–02.

## Competing interests

The authors declare that they have no competing interests.

## Authors' contributions

The study was proposed by CM. LH and SD carried out the preliminary analyses and all authors discussed the findings. Final analyses were carried out by LH and KSJ. KSJ wrote the first draft of the manuscript and all authors contributed to the intellectual content. All authors approved the final version of the manuscript.

## Pre-publication history

The pre-publication history for this paper can be accessed here:


